# Supplementary material for: Predicted deleterious mutations reveal the genetic architecture of male reproductive success in a lekking bird
Source: Nat Ecol Evol. 2025 Aug 11;9(10):1924–37. doi: 10.1038/s41559-025-02802-8 (PMC12507671; doi:10.1038/s41559-025-02802-8)
Supplement: Supplementary file 1 — Supplementary Results and Discussion, Methods, Figs. 1–3 and Tables 1–15. [file 41559_2025_2802_MOESM1_ESM.pdf]

nature ecology & evolution

Supplementary information

https://doi.org/10.1038/s41559-025-02802-8

Predicted deleterious mutations reveal the genetic architecture of male reproductive success in a lekking bird

In the format provided by the authors and unedited

**Supplementary Results and Discussion**	**Pages 2 – 5**
**Supplementary Methods**	**Pages 5 – 10**
**Supplementary Figures**	**Pages 11 – 13**
**Supplementary Tables**	**Pages 14 – 30**
**References**	**Pages 31 – 37**

Supplementary Results and Discussion

Population and relatedness structure

To provide context for our study, we first characterised the genetic structure of the black grouse population and tested for the presence of close kin both within and among the lekking sites (Supplementary Fig. 1a). Genetic differentiation was overall rather weak, with no pairwise comparisons of lekking sites yielding F_{ST} values that differed significantly from zero (Supplementary Table 12). However, there was a hint of population structure along a west-east axis, with KUM and NYR being the most differentiated lekking sites (Supplementary Fig. 1b). These findings are consistent with previous studies revealing weak to moderate population structure among black grouse leks[1–3] and reflect the interplay between female dispersal and male site fidelity[1,4,5]. Accordingly, no significant differences were found among the lekking sites in individual mutation loads and only one pair of lekking sites differed significantly in F_{ROH} (Supplementary Table 13).

To characterise the relatedness structure of our dataset, we calculated R0, R1 and KING-robust kinship values for all pairwise combinations of individuals and visualised the results by plotting R1 against KING-robust kinship (Supplementary Fig. 1c). Pairs of individuals were assigned to specific relatedness categories by computing Z scores and comparing these with the inference criteria of Manichaikul et al.[6]. Among the 17,949 pairwise comparisons, the majority were unrelated pairs (17,483 = 97.4%), with only 466 pairs (2.6%) being identified as close kin. The close kin pairs were comprised of 27 full siblings (5.8%), 60 parent-offspring pairs (12.9%), 176 second-degree relatives (37.8%) and 203 third-degree relatives (43.6%). Out of 190 individuals, 104 had at least one first-degree relative (full sibling or parent-offspring) in the population, 124 had at least one second-degree relative, and 143 had at least one third-degree relative. The proportion of relatives sampled at the same lekking site was highest for full siblings and lowest for third-degree relatives (Supplementary Fig. 1d). The presence of close male kin on black grouse leks has been described previously[7] and arises due to strong male philopatry[1,4,5]. While the risk of inbreeding is reduced by female-biased dispersal[8], consanguineous matings may still occur as older females have a greater chance of mating with philopatric male kin[9].

Differences between GERP and SnpEff

The nearly two orders of magnitude difference in the absolute number of mutations identified as deleterious by GERP (n = 413,489) and SnpEff (n = 5,341) can be attributed to conceptual and methodological differences between the two approaches[10,11]. SnpEff restricts its annotation of high impact variants to genes, where mutations are more likely to affect transcripts and protein products[11]. By contrast, GERP scores are calculated across the entire genome, including intergenic regions[10]. Furthermore, SnpEff evaluates the likely impact of mutations on amino acid sequences[11], whereas GERP quantifies evolutionary conservation without regard to a mutation's functional effect on protein structure and function[10]. While this disparity in the number of identified mutations is therefore expected, it raises an important question: are both approaches are equally effective at identifying mutations that reduce fitness?

Moreover, the absolute number of deleterious mutations identified in a population can be influenced by numerous biological and methodological factors. Species- and population-specific characteristics such as the mutation rate, effective population size, strength of selection, inbreeding levels and purging directly affect the abundance of deleterious variants[12–14]. Methodological aspects including sample size, sequencing depth and read quality also determine the number of reliably called SNPs[15,16], while downstream analytical choices such as SNP filtering criteria and the choice of the GERP score threshold used to classify a mutation as deleterious substantially affect the final count of predicted deleterious mutations. However, standardising this threshold across species is difficult because the range of GERP scores is determined by the phylogenetic tree used for GERP score computation[10]. It is likely that these and possibly other factors contribute to the high proportion of mutations annotated as deleterious in the black grouse (5.9% of all SNPs) when compared to some[10,14] but not all[17] previous vertebrate studies.

We found a stronger negative effect of the total GERP load on LMS compared to the SnpEff load, which may reflect the distinct properties of those mutations identified by each prediction approach. High GERP scores are considered strong indicators of deleterious mutations under purifying selection[18]. By contrast, although mutations classified as high impact by SnpEff, such as LOF mutations, are also expected to have negative effects on fitness[19,20], some may instead be adaptive or neutral[21–23], might not necessarily be relevant to the trait in question[11], or their phenotypic effects might not be as strong as expected due to alternative splicing or because the function of the focal protein might be rescued by another protein[24].

Additionally, the effect sizes of the total GERP and SnpEff load may be influenced by the differences in the number of mutations contributing to each load estimate. To investigate whether the observed effect sizes of the total GERP load and the total SnpEff load are driven by differences in the effect sizes of individual mutations or by differences in the number of contributing mutations, we performed random sampling of equal-sized subsets of mutations as described in the Supplementary Methods. First, we compared the effect sizes of the total GERP load and the total SnpEff load after randomly sampling 5,000 subsets of 1,000 mutations for both prediction approaches. The effect sizes of the total load on LMS were similar for GERP and SnpEff (median β estimate = -0.05 and -0.11 respectively; Supplementary Fig 3a, b), suggesting that the stronger association between the total GERP load and LMS in the full dataset is mainly due to the larger number of identified mutations, which collectively provide greater statistical power to detect an effect.

To compare the effect sizes of the four genomic regions (promoter, TSS, intron, exon), we repeated our analyses using random subsets of 500 mutations per region. We found that the effect sizes of the total load on LMS were substantially more negative for mutations with GERP scores ≥ 4 in TSSs (median β estimate = -0.21) and high impact SnpEff mutations in promotors (median β estimate = -0.29) compared to mutations in other genomic regions (median β estimates for GERP: promoters = -0.06; introns = -0.04, exons = 0.05; median β estimates for SnpEff: TSSs = -0.06, introns = -0.06, exons = -0.07; Supplementary Fig 3c, d). This provides further evidence for deleterious mutations in these critical regulatory regions having disproportionately negative fitness effects.

Biological processes

While deleterious mutations could in principle affect fitness via diverse biological processes, previous research has identified specific biological processes that are likely to be particularly important for sexual signaling and sexual selection in black grouse and other animals [25–28]. Based on this, we hypothesised that male reproductive success in black grouse would be especially sensitive to mutations affecting androgen metabolism (including testosterone which strongly correlates with sexual trait expression in black grouse[28,29]), cellular respiration (which is crucial for energetically costly behaviours such as lekking[30]), developmental growth (which predicts survival[31] and territory-holding ability in black grouse[30]), immune function (hypothesized to link testosterone-dependent sexual traits to honest signaling[25,26]), muscle tissue development (greater glycogen storage in muscles allows higher display rates in male

black grouse[32,33]) and responses to oxidative stress (which influence carotenoid and melanin-based sexual ornaments[27]). For details of our hypotheses and rationale, see Supplementary table 5.

To test these hypotheses, we used gene ontology (GO) annotations to identify subsets of deleterious mutations within genes associated with these six biological processes (see Supplementary Methods and Supplementary Table 5 for details). We then computed the total load for each GO term and prediction approach separately for promoters, introns and exons. These values were then used as predictor variables in separate Bayesian GLMMs of LMS, following the same model structure described above. This hypothesis-driven approach, akin to a candidate gene framework[34], tests for associations between our six focal GO terms and male reproductive success, but does not allow inference about other biological processes not included in our *a priori* hypotheses.

Overall, while the effect of the total load on LMS varied in both direction and significance depending on the GO term and genomic region, three distinct patterns emerged. First, GERP mutations in promotor regions were consistently negatively associated with LMS across all GO terms (Extended Data Fig. 5, Supplementary Table 6), reinforcing our earlier finding that deleterious mutations in promoter regions tend to reduce fitness. Second, for androgen metabolism, immune response and response to oxidative stress, GERP mutations in at least two genomic regions were negatively associated with LMS (Extended Data Fig. 5, Supplementary Table 6). Third, SnpEff mutations in immune-related genes had consistently negative effects on LMS across all three genomic regions (Extended Data Fig 6, Supplementary Table 7). These findings are consistent with the strong selective pressures exerted by pathogens on immune genes[35], the well-established links between sexual traits and immune function[36–38], the importance of androgens for sexual trait expression[28,29] and the detrimental effects of oxidative stress on numerous life-history traits[39].

Supplementary Methods

Library preparation

Library preparation of the 190 whole genomic extracts was performed at the Beijing Genomics Institute (BGI) using the BGI Optimal DNA Library Prep Kit (BGI – Shenzhen China). Genomic DNA Fragments were size-selected using magnetic beads subjected to end repair to generate blunt-ended DNA. A single adenosine was added to the 3’ ends via an A-tailing

reaction, followed by adapter ligation. The resulting libraries were PCR amplified and the double stranded library products were denatured to generate single stranded library products. A circularization reaction was then used to generate single stranded circularized DNA products, while excess single stranded linear DNA was removed through digestion. The resulting single stranded circularized libraries were amplified using phi29 and rolling circle amplification to generate a DNA nanoball (DNB) that carries approximately 300 copies of the initial single-stranded library molecules.

Reference genome

For this study, we scaffolded a black grouse reference genome assembled by the 10K Bird Project (B10K)[40,41]. The black grouse individual originated from Nordland, Norway and was preserved at the Natural History Museum of Denmark. B10K constructed a single-tube long fragment read co-barcoded (stLFR) library which was subsequently 100bp paired-end sequenced on a DNBseq platform at the Beijing Genomics Institute (BGI). Raw reads with missing data (>10%), low quality (>40% bases with Phred score <= 10) or below expected insert sizes were excluded and PCR duplicates were filtered out using SOAPfilter2 v2.2[42]. GapCloser v1.12[43] was used to close gaps within scaffolds. The resulting B10K assembly had a total length of 1,002,957,384 bp and consisted of 26,930 scaffolds with an N50 of 5,658,217 bp (see Supplementary Table 14 for details).

To improve contiguity, we scaffolded the B10K assembly using HiRise together with Cantata Bio. They used 200 ml of blood to prepare an Omni-C library for genome scaffolding with HiRise. First, chromatin in the nucleus was fixed with formaldehyde[44]. DNase I was then used to extract and digest the chromatin, after which the ends were repaired and ligated to a biotinylated bridge adapter. The DNA was subsequently purified and unligated fragments were discarded. A library was then generated using NEBNext Ultra enzymes and Illumina-compatible adapters. Using streptavidin beads, biotin-containing fragments were isolated and the sequences were duplicated in a polymerase chain reaction (PCR). Prior to deep sequencing, the B10K assembly was used to quality check the OmniC library. Finally, the sequencing was performed using an Illumina HiSeqX platform with a target coverage of 30x and the resulting reads were quality filtered (MQ > 50) and used to scaffold both pseudo-haplotypes with Cantata Bio's HiRise software[44]. The contiguity of the resulting reference genome was considerably improved, with the scaffold N50 increasing over ten-fold to 69,550,540 (Supplementary Table 14). Lastly, we identified the scaffold corresponding to the Z chromosome by aligning the

chicken Z chromosome (NCBI RefSeq assembly GCF_016699485.2) to the black grouse reference genome using BLAST v2.12.0[45]. We identified a long scaffold that showed 83% identity to the chicken Z chromosome, which we assigned as the black grouse Z chromosome.

Genome annotation

To annotate the scaffolded reference genome, we generated transcriptomic data from an 11–14 day old black grouse embryo obtained from a captive breeding facility in the Netherlands. Total RNA was extracted from a mixture of randomly selected embryo sections using the Qiagen RNeasy Plus Kit. The extract was treated with DNase and the RNA was cleaned using AMPure beads and the QIAGEN FastSelect HMR RNA depletion kit. The library was prepared by Genewiz Multiomics & Synthesis Solutions using the NEBNext Ultra II RNA Library Prep Kit and 150 bp paired-end sequenced on an Illumina NovaSeq6000 platform.

The genome annotation was performed by Cantata Bio using the resulting RNA sequencing data together with publicly available RNA sequencing data (NCBI SRA Bioproject SRP006680). Repeat families in the genome were identified *de novo* and classified using RepeatModeler v2.0.1[46]. The output from RepeatModeler was then used to identify and mask repeated segments in the genome annotation file using RepeatMasker v4.1.0[47]. Two initial *ab initio* gene models were trained with the coding sequences of the chicken (*Gallus gallus*), Japanese quail (*Coturnix japonica*), rock ptarmigan (*Lagopus muta*) and wild turkey (*Meleagris gallopavo*) using AUGUSTUS v2.5.5[48] and SNAP v2006-07-28[49] respectively. The AUGUSTUS predictions were optimised in six rounds. RNA-Seq reads were aligned to the reference genome using STAR v2.7[50] and intron hints were generated with the bam2hints tools within AUGUSTUS. Subsequently, we used MAKER[51], SNAP and AUGUSTUS (with intron-exon boundary hints provided from the RNA-Seq data) to predict genes in the repeat-masked reference genome. To help guide the gene prediction process, Swiss-Prot peptide sequences from the UniProt database were downloaded and used in combination with the protein sequences from the avian species described above to generate peptide evidence in the Maker pipeline. The final gene sets comprised only genes that were predicted by both SNAP and AUGUSTUS. The quality of the predictions was assessed using AED scores generated for predicted genes by MAKER. Genes were further characterised for their putative function by performing a BLAST[45] search of the peptide sequences against the UniProt database. Transfer RNAs were predicted using tRNAscan-SE v2.05[52].

Population structure and relatedness

We used principal component analysis (PCA) to characterise population genetic structure. For this analysis, PLINK v1.90[53] was used to produce a stringently filtered SNP dataset from which the following were excluded (i) strongly linked SNPs, where linkage disequilibrium (LD) was computed in window sizes of 50 base pairs with shifts of five SNPs with a variance inflation factor threshold of two (--indep 50 5 2); (ii) SNPs deviating significantly from Hardy-Weinberg equilibrium (HWE) with an alpha level of 0.001 (--hwe 0.001); and (iii) SNPs with a minor allele frequency (MAF) below 0.01 (--maf 0.01). The PCA was implemented using PLINK (--pca) and the results were visualized with the R tidyverse package set v1.3.1[54], including dplyr v1.1.4 and ggplot2 v3.5.1.

Next, we quantified genetic differentiation by calculating F_{ST} values between each pair of lekking sites based on the stringently filtered dataset described above. F_{ST} values were computed per SNP using VCFtools v0.1.17[55] and then averaged across loci. To test whether the mean F_{ST} values were significantly different from zero, we computed 1,000 bootstrap replicates by resampling the pairwise F_{ST} values across loci with replacement using the boot package v.1.3.28[56]. Finally, we calculated the absolute differences between the resampled mean F_{ST} values and the mean F_{ST} of the original data, summed these values and divided them by the total number of bootstrap replicates to obtain the p-value, which represents the probability of observing a mean F_{ST} different from zero.

We used the same stringently filtered dataset to infer patterns of pairwise genomic relatedness among individuals following the workflow of Humble *et al.*[57]. NgsRelate v2[58] was used to compute three relatedness indices: KING-robust kinship, R0 and R1[59]. The KING-robust kinship estimate can be used to distinguish between different levels of relatedness when allele frequencies are unknown and is robust to population structure[6]. The R0 and R1 statistics specify whether zero or one copies of an allele are shared, respectively. Different categories of relatedness are associated with non-overlapping ranges of R1, R0 and KING-robust kinship values.

To allocate pairs of individuals to specific relationship categories, we calculated the relatedness coefficients $\hat{p}$, Z0, Z1 and Z2 using the -genome function in PLINK[53]. $\hat{p}$ is the overall proportion of the genome that is identical by descent between any pair of individuals. Z0, Z1 and Z2 are coefficients that estimate the proportion of the genome for which zero, one or two

alleles of a pair of individuals are identical by descent, respectively. We used the method described in Manichaikul *et al.*[6] to assign relationship categories to each pair of individuals based on theoretical thresholds of the various relatedness coefficients. Pairs of individuals were assigned to one of five relatedness categories: parent-offspring, full siblings, second-degree relatives (e.g. half-siblings and grandparents-grandchildren), third-degree relatedness (e.g. cousins) or unrelated individuals. To allow minor deviations from the theoretical expectations for certain relationships, we classified pairs of individuals as falling within a given relatedness category if they were within 0.01 of the respective inference threshold, following Waples *et al.*[59]. Those pairs that did not fall within the theoretical ranges of any category including this additional margin were classified as "unknown".

Controlling for the number of mutations

We compared the effect sizes of the total GERP load and the total SnpEff load while controlling for the number of mutations. We randomly sampled 5,000 subsets of 1,000 mutations separately for mutations with GERP scores ≥ 4 and high impact SnpEff mutations. For each subset, we then calculated the total load and fitted this as a predictor variable in a GLMM of LMS. We included core versus non-core fitted as a fixed effect and lekking site as a random effect. However, to decrease computational demand, these models were implemented using a frequentist approach in the R package glmmTMB v1.1.10[60]. We also repeated the above steps to compare the effect sizes of mutations residing in the four genomic regions (promoters, TSSs, introns and exons) separately for GERP and SnpEff. Because there were fewer mutations on average in each of the four regions, we only selected 500 mutations for each random sample. We calculated the tolerance intervals of the β estimates, which indicate the proportion of values covered by the interval at a given confidence level, using the R package tolerance v3.0.0 [61].

Testing for the effects of mutations across biological pathways

We hypothesised that mutations affecting six biological processes could be particularly relevant for reproductive success in black grouse males (Supplementary Table 5). To test this, we used gene ontology (GO) annotations to identify subsets of deleterious mutations associated with each biological process. A list of genes corresponding to each GO term along with their descriptions[62] was obtained using AmiGO version 2.5.17[63] (release date 2025-02-08). For each GO term, we then extracted the deleterious mutations located in the promoter regions, introns and exons of the associated genes (Supplementary Table 15). Mutations at the TSS were not analysed separately, as this region is considered part of the promoter and contained relatively

few mutations for each GO term. To ensure the robustness of the analysis, we only included GO-specific subsets with at least 15 mutations, which resulted in the exclusion of one GO term (androgen metabolism) for SnpEff. We then calculated the total GERP load and total SnpEff load separately for each subset of mutations and for each of the three genomic regions and constructed Bayesian GLMMs of LMS as described above, one for each GO term, genomic region and prediction approach. The total mutation loads were again z-transformed and the same controlling variables and random effect structure was used as described above.

Supplementary Figures

Supplementary Fig. 1. Genetic and relatedness structure of the black grouse study population in central Finland. (a) Geographical locations of the lekking sites, with circle sizes being proportional to the number of sampled lekking males (total *n* = 190) as shown in the legend. Abbreviations: KUM = Kummunsuo, NYR = Nyrölä, SAA = Saarisuo, LEH = Lehtosuo, TEE = Teerisuo; (b) Results of the principal component analysis, with the lekking sites colour coded as shown in the legend; (c) R1 coefficients plotted against KING-robust kinship coefficients for all individual pairwise comparisons. The colours of the points indicate relationship categories inferred by comparing PLINK Z scores with the inference criteria derived from Manichaikul *et al.*[6] as shown in the legend; (d) A breakdown of the relatedness structure of the population divided into comparisons within and among lekking sites.

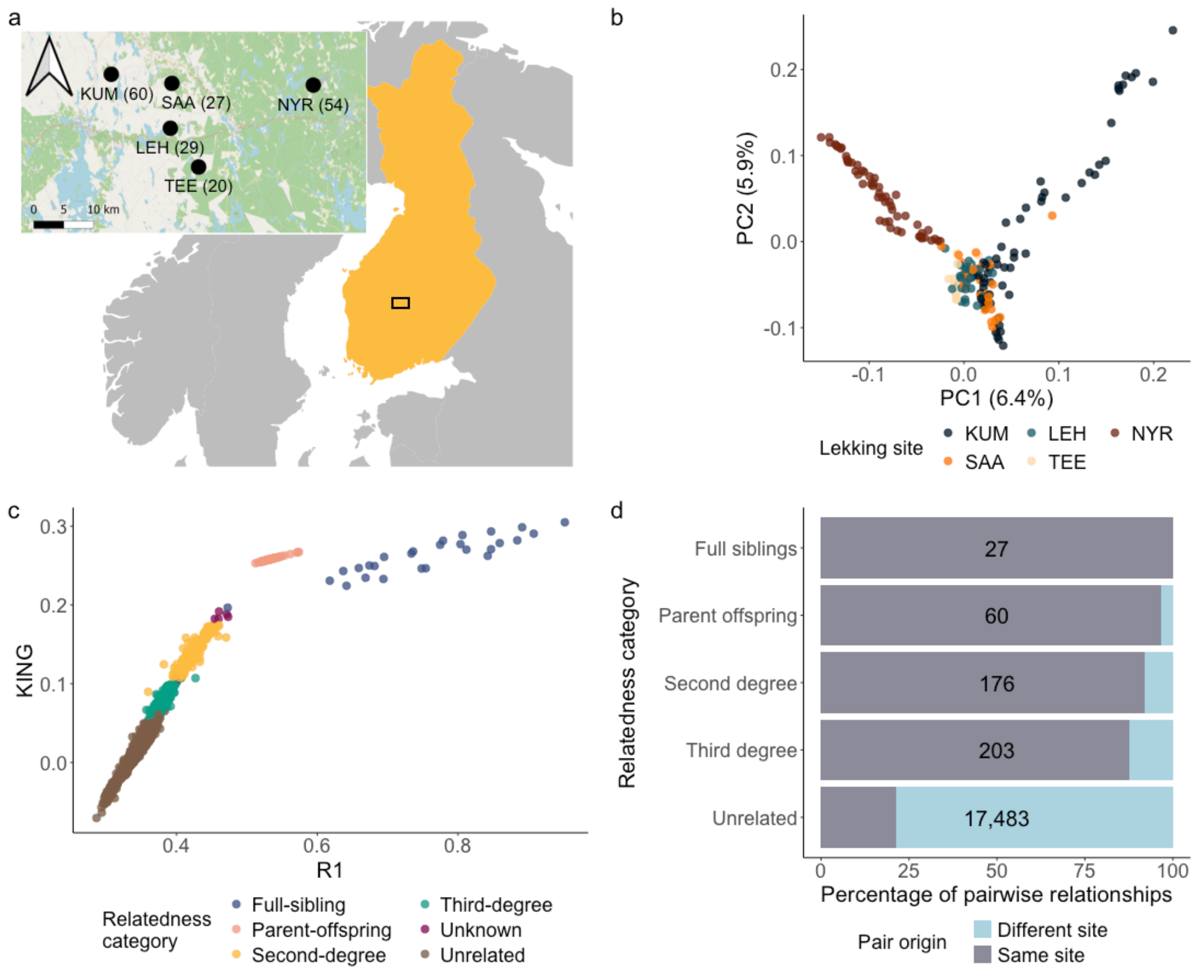

Supplementary Fig. 2. Phylogenetic tree used for the calculation of GERP scores. Shown is an unrooted phylogenetic tree consisting of 74 avian species that was used for calculating GERP scores. Different avian clades are colour-coded as shown in the legend.

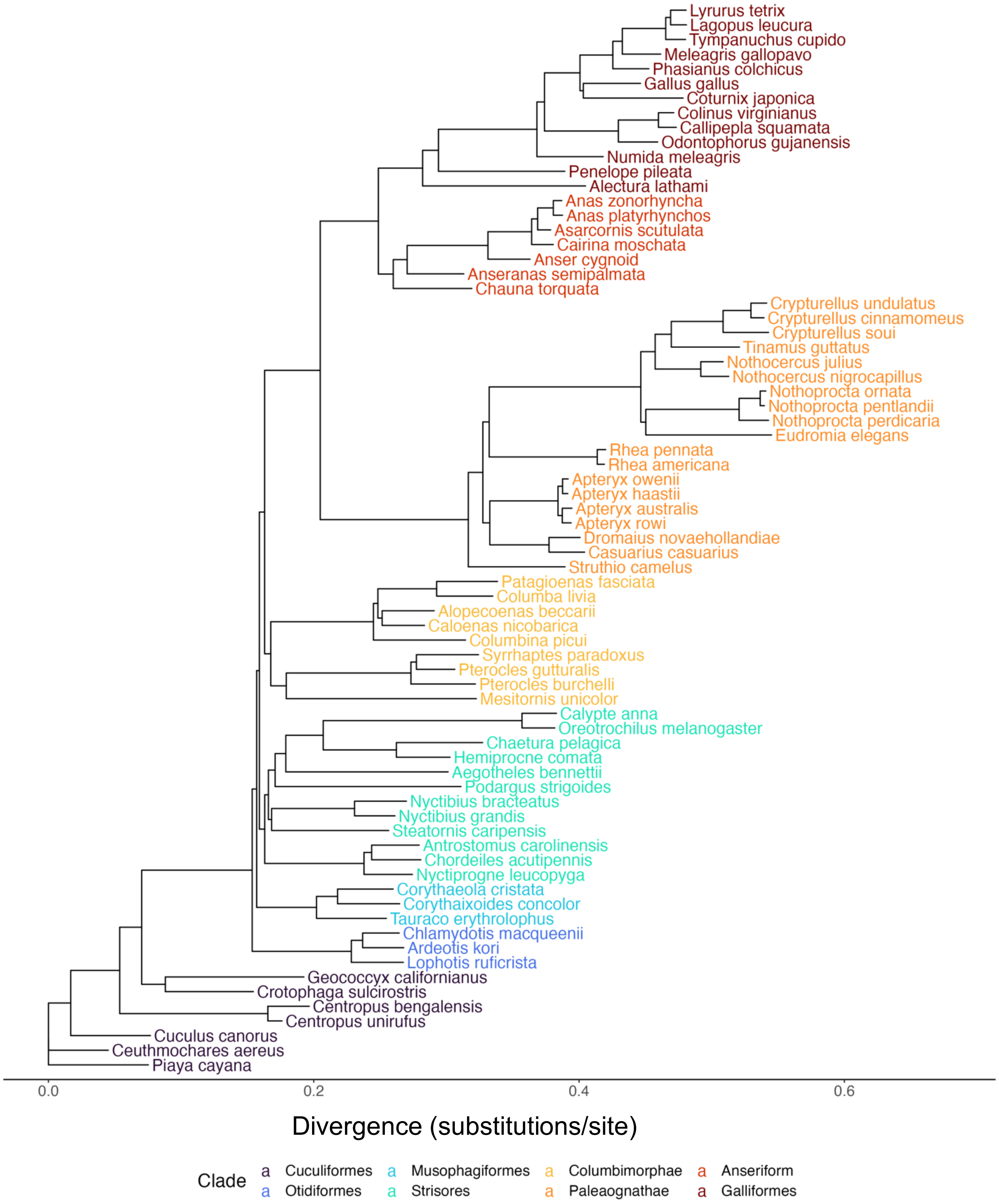

Supplementary Fig. 3. Distribution of fitness effects based on random subsamples of deleterious mutations. Shown are histograms of the standardised β estimates of genomic mutation load components based on randomly selected subsets of mutations on lifetime mating success. For (a) the total GERP load and (b) the total SnpEff load, we took 5,000 randomly selected subsets of 1,000 mutations. For (c) the total GERP load subsetted into four genomic regions and (d) the total SnpEff load subsetted into four genomic regions, we took 5,000 randomly selected subsets of 500 mutations. The white circles represent the mean β estimates, the thick black lines the 80% tolerance intervals (alpha = 0.05) and the thin black lines the 95% tolerance intervals (alpha = 0.05). The GERP load is shown in red and the SnpEff load is shown in dark grey.

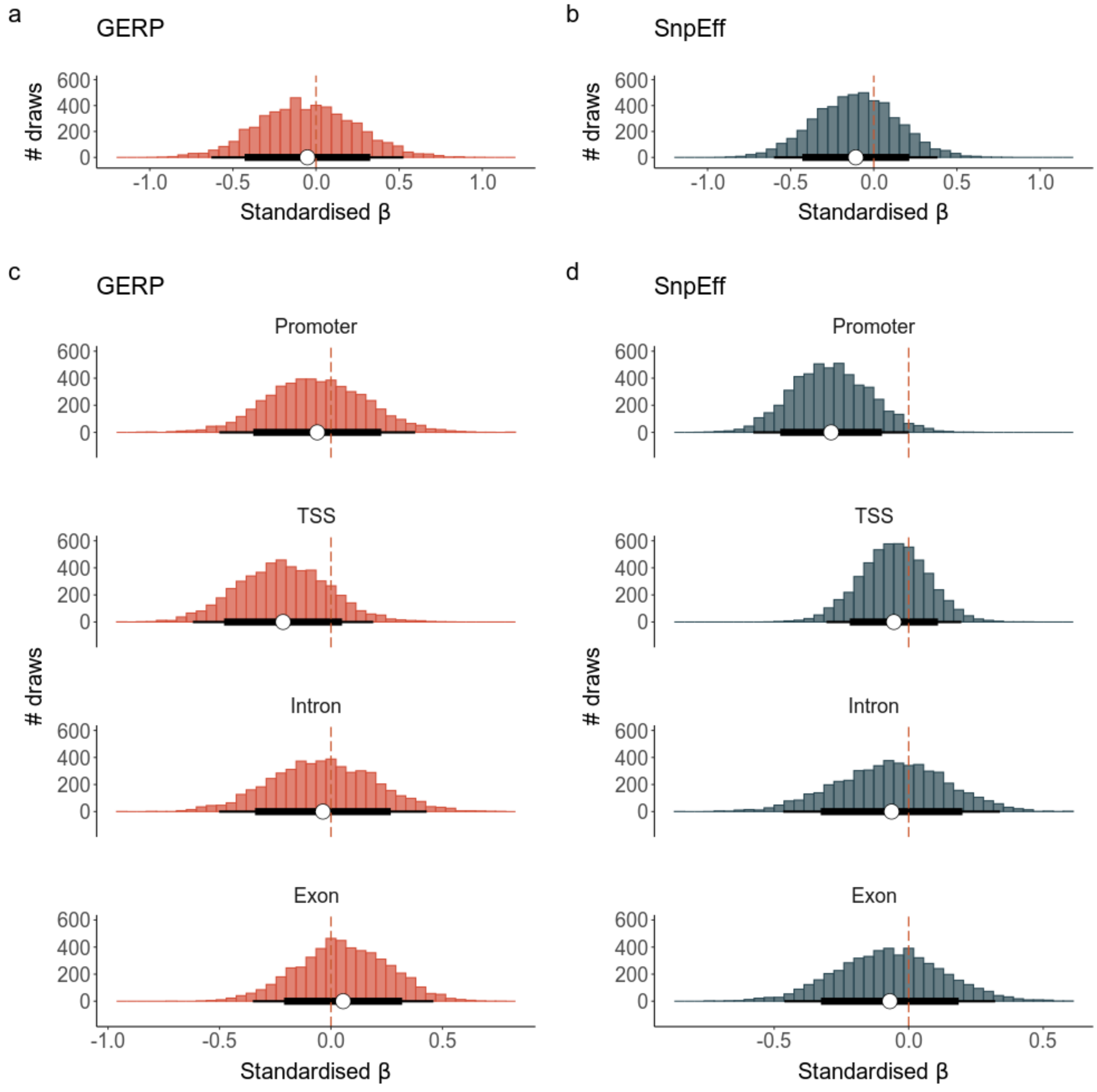

Supplementary Tables

Supplementary Table 1. Outputs of Bayesian GLMMs of lifetime mating success (LMS). Shown are point estimates, credible intervals (CIs; 95% and 80%) and R^2 values of the standardised β estimates of the genomic predictors listed in the left column on LMS. The conditional R^2 refers to the variance explained by the fixed and random effects, whereas the marginal R^2 refers to the variance explained only by the fixed effects.

**Predictor**	**Median**	**95% CI (lower, upper)**	**80% CI (lower, upper)**	**Conditional R^2 [95%CI]**	**Marginal R^2 [95%CI]**
Total GERP load	-0.21	-0.27, -0.14	-0.25, -0.17	0.07 [0.04, 0.11]	0.02 [$3.64e^{-5}$, 0.06]
Total SnpEff load	-0.11	-0.18, -0.04	-0.16, -0.06	0.05 [0.03, 0.08])	0.01 [$3.69e^{-5}$, 0.03]
F_{ROH}	-0.14	-0.20, -0.07	-0.23, -0.05	0.05 [0.03, 0.08]	$8.00e^{-3}$ [$4.57e^{-6}$, 0.03]
Homozygous GERP load	-0.57	-0.76, -0.39	-0.70, -0.45	0.07 [0.04, 0.11]	0.02 [$1.68e^{-4}$, 0.06]
Heterozygous GERP load	-0.60	-0.78, -0.41	-0.72, -0.48		
Homozygous SnpEff load	-0.09	-0.17, -0.01	-0.15, -0.04	0.05 [-.03, 0.08]	0.01 [$2.87e^{-4}$, 0.03]
Heterozygous SnpEff load	-0.15	-0.24, -0.06	-0.21, -0.09		

Supplementary Table 2. Full outputs of Bayesian GLMMs of lifetime mating success (LMS) as a function of the total GERP load, the total SnpEff load and F_{ROH}. Shown are point estimates and credible intervals (95% and 80%) of the intercepts, standardised β estimates, standard deviations (SD) and zero-inflation factors. For the parameter 'core versus non-core', the β estimates of the non-core males relative to the core males are shown. Because all of the non-genomic terms are identical across the LMS models, we only report the full model outputs of these exemplary models in order to avoid redundancy. The full outputs of all of the other models can be found in the github repository (see the data availability statement for details).

Model	Parameter	Median	95% CI (lower, upper)	80% CI (lower, upper)
Total GERP load	Intercept	1.73	0.91, 2.36	1.31, 2.08
	β of the total GERP load	-0.21	-0.27, -0.14	-0.25, -0.17
	β of core versus non-core	0.33	0.14, 0.51	0.20, 0.45
	SD intercept of lek site	0.59	0.29, 1.86	0.37, 1.14
	Zero inflation	0.49	0.42, 0.57	0.45, 0.54
	Intercept Kummunsuo	-0.05	-0.68, 0.77	-0.41, 0.38
	Intercept Lehtosuo	-0.43	-1.16, 0.33	-0.84, -0.02
	Intercept Nyrölä	-0.19	-0.82, 0.61	-0.54, 0.26
	Intercept Saarisuo	0.79	0.17, 1.59	0.43, 1.23
	Intercept Teerisuo	0.10	-0.52, 0.93	-0.26, 0.51
Total SnpEff load	Intercept	1.76	1.09, 2.34	1.4, 2.07
	β of the total SnpEff load	-0.11	-0.18, -0.04	-0.16, -0.06
	β of core versus non-core	0.43	0.24, 0.61	0.30, 0.55
	SD intercept of lek site	0.52	0.25, 1.60	0.32, 1.00
	Zero inflation	0.50	0.42, 0.57	0.44, 0.54
	Intercept Kummunsuo	-0.07	-0.67, 0.61	-0.40, 0.28
	Intercept Lehtosuo	-0.27	-0.92, 0.40	-0.64, 0.11
	Intercept Nyrölä	-0.25	-0.87, 0.41	-0.59, 0.10
	Intercept Saarisuo	0.69	0.10, 1.34	0.36, 1.07
	Intercept Teerisuo	0.03	-0.56, 0.71	-0.31, 0.40

F_{ROH}	Intercept	1.76	0.89, 2.36	1.39, 2.10
	β of F_{ROH}	-0.14	-0.23, -0.05	-0.20, -0.07
	β of core versus non-core	0.38	0.19, 0.56	0.26, 0.50
	SD intercept of lek site	0.54	0.26, 1.70	0.32, 1.06
	Zero inflation	0.50	0.43, 0.57	0.45, 0.54
	Intercept Kummunsuo	-0.07	-0.67, 0.77	-0.42, 0.32
	Intercept Lehtosuo	-0.34	-1.02, 0.48	-0.72, 0.07
	Intercept Nyrölä	-0.24	-0.87, 0.62	-0.60, 0.14
	Intercept Saarisuo	0.69	0.10, 1.60	0.36, 1.09
	Intercept Teerisuo	0.09	-0.56, 0.99	-0.26, 0.50

Supplementary Table 3. Outputs of Bayesian GLMMs of lifetime mating success stratified by genomic region for deleterious GERP mutations (GERP scores ≥ 4). Shown are point estimates, credible intervals (95% and 80%) and R^2 values of the standardised β estimates of the total GERP load calculated per genomic region. The conditional R^2 refers to the variance explained by the fixed and random effects, whereas the marginal R^2 refers to the variance explained only by the fixed effects.

**Region**	**Median**	**95% CI (lower, upper)**	**80% CI (lower, upper)**	**Conditional R^2 [95%CI]**	**Marginal R^2 [95%CI]**
Promoter	-0.18	-0.26, -0.09	-0.23, -0.13	0.07 [0.04, 0.11]	0.01 [4.18e^{-5}, 0.04]
TSS	-0.27	-0.35, -0.20	-0.32, -0.22	0.07 [0.04, 0.11]	0.03 [1.00e^{-3}, 0.07]
Intron	-0.29	-0.37, -0.21	-0.35, -0.23	0.06 [0.04, 0.09]	0.03 [2.0e^{-3}, 0.06]
Exon	0.23	0.15, 0.31	0.18, 0.29	0.07 [0.04, 0.11]	0.02 [3.00e^{-4}, 0.05]

Supplementary Table 4. Outputs of Bayesian GLMMs of lifetime mating success stratified by genomic region for high impact SnpEff mutations. Shown are point estimates, credible intervals (95% and 80%) and R^2 values of the standardised β estimates of the total SnpEff load calculated per genomic region. The conditional R^2 refers to the variance explained by the fixed and random effects, whereas the marginal R^2 refers to the variance explained only by the fixed effects.

**Region**	**Median**	**95% CI (lower, upper)**	**80% CI (lower, upper)**	**Conditional R^2 [95%CI]**	**Marginal R^2 [95%CI]**
Promoter	-0.26	-0.34, -0.18	-0.31, -0.20	0.06 [0.04, 0.10]	0.02 [1.00e^{-3}, 0.06]
TSS	-0.04	-0.12, 0.04	-0.09, 0.01	0.05 [0.03, 0.09]	0.05 [1.56e^{-5}, 0.02]
Intron	-0.08	-0.15, 0.01	-0.13, -0.02	0.05 [0.03, 0.08]	0.01 [8,93e^{-5}, 0.03]
Exon	-0.06	-0.20, -0.03	-0.17, -0.06	0.05 [0.03, 0.08]	0.01 [6.60^{-6}, 0.03]

Supplementary Table 5. List of GO terms hypothesized to be important for male reproductive success in black grouse. For each GO term, we provide its description from AmiGO[63] and the rationale for why deleterious mutations in genes associated with this term may impact male mating success in black grouse.

GO term	GO Accession	Description	Rationale	Hypothesis
Androgen metabolic process	GO:0008209	The chemical reactions and pathways involving androgens, C19 steroid hormones that can stimulate the development of male sexual characteristics.	Testosterone, an androgen, strongly correlates with lek centrality, red eye comb size[29], and mating success[28] in male black grouse.	Deleterious mutations that reduce androgen production will be detrimental to male LMS.
Cellular respiration	GO:0045333	The enzymatic release of energy from inorganic and organic compounds (especially carbohydrates and fats) which either requires oxygen (aerobic respiration) or does not (anaerobic respiration).	Lekking behaviour is energetically costly[30] and efficient cellular respiration may be important for sustaining such demanding behaviour.	Deleterious mutations that affect the efficiency of cellular respiration will be detrimental to male LMS.
Developmental growth	GO:0048589	The increase in size or mass of an entire organism, a part of an organism or a cell, where the increase in size or mass has the specific outcome of the progression of the organism over time from one condition to another.	Body mass in Galliformes is positively correlated with the growth of legs, wings and the sternum throughout development[64]. Body mass in black grouse is a predictor of chick[31] and yearling survival[31] and correlates with yearling territoriality[30].	Deleterious mutations that reduce developmental growth will be detrimental to male LMS.

Immune response	GO:0006955	Any immune system process that functions in the calibrated response of an organism to a potential internal or invasive threat.	Sexual traits are expected to be honest signals of quality due to the impairment of immune function associated with elevated testosterone levels[25,26]. Indeed, stabilizing selection acts on immunity in black grouse males and has consequences for sexual trait expression[65].	Deleterious mutations that cause a suboptimal immune response will be detrimental to male LMS.
Muscle tissue development	GO:0060537	The progression of muscle tissue over time, from its initial formation to its mature state. Muscle tissue is a contractile tissue made up of actin and myosin fibers.	Individuals with greater glycogen storage in muscles (e.g. due to greater muscle mass) may be able to maintain higher display rates and store more energy[32,33]. Higher display rates increase black grouse reproductive success[66,67].	Deleterious mutations that impact muscle synthesis and maintenance will be detrimental to male LMS.
Response to oxidative stress	GO:0006979	Any process that results in a change in state or activity of a cell or an organism (in terms of movement, secretion, enzyme production, gene expression, etc.) as a result of oxidative stress, a state often resulting from exposure to high levels of reactive oxygen species, e.g. superoxide anions, hydrogen peroxide (H2O2), and hydroxyl radicals.	Oxidative stress can affect investment in growth and/or sexual traits[68], especially the expression of carotenoid and melanin-based ornamental traits[27].	Deleterious mutations that impact susceptibility to oxidative stress will be detrimental to male LMS.

Supplementary Table 6. Outputs of Bayesian GLMMs of lifetime mating success stratified by genomic region and GO term for deleterious GERP mutations (GERP scores ≥ 4). Shown are point estimates and credible intervals (95% and 80%) of the standardised β estimates of the total GERP load calculated separately for each GO term and stratified by genomic region (promoters, introns and exons).

**Genomic region**	**GO term**	**Median**	**95% CI (lower, upper)**	**80% CI (lower, upper)**
Promoters	Androgen metabolism	-0.33	-0.40, -0.25	-0.38, -0.28
	Cellular respiration	-0.25	-0.34, -0.16	-0.30, -0.19
	Developmental growth	-0.22	-0.30, -0.15	-0.27, -0.17
	Immune response	-0.23	-0.30, -0.15	-0.28, -0.18
	Muscle tissue development	-0.09	-0.17, -0.01	-0.14, -0.04
	Response to oxidative stress	-0.11	-0.19, -0.02	-0.16, -0.05
Introns	Androgen metabolism	0.41	0.32, 0.49	0.35, 0.46
	Cellular respiration	0.33	0.23, 0.43	0.27, 0.40
	Developmental growth	0.09	$1.46e^{-3}$, 0.18	0.03, 0.15
	Immune response	-0.29	-0.38, -0.21	-0.35, -0.24
	Muscle tissue development	0.08	0.01, 0.15	0.03, 0.13
	Response to oxidative stress	-0.03	-0.12, 0.06	-0.09, 0.03
Exons	Androgen metabolism	-0.17	-0.25, -0.10	-0.23, -0.12
	Cellular respiration	0.01	-0.06, 0.09	-0.03, 0.07
	Developmental growth	0.05	-0.04, 0.13	-0.01, 0.10
	Immune response	0.14	0.06, 0.23	0.08, 0.20
	Muscle tissue development	-0.02	-0.10, 0.07	-0.07, 0.04
	Response to oxidative stress	-0.17	-0.25, -0.09	-0.22, -0.11

Supplementary Table 7. Outputs of Bayesian GLMMs of lifetime mating success stratified by genomic region and GO term for high impact SnpEff mutations. Shown are point estimates and credible intervals (95% and 80%) of the standardised β estimates of the total SnpEff load calculated separately for each GO term and stratified by genomic region.

**Gene region**	**GO term**	**Median**	**95% CI (lower, upper)**	**80% CI (lower, upper)**
Promoters	Cellular respiration	$8.54e^{-5}$	-0.09, 0.09	-0.06, 0.06
	Developmental growth	-0.05	-0.14, 0.03	-0.11, $2.73e^{-3}$
	Immune response	-0.25	-0.34, -0.17	-0.31, -0.20
	Muscle tissue development	0.05	-0.03, 0.13	$1.82e^{-4}$, 0.10
	Response to oxidative stress	0.11	0.03, 0.19	0.06, 0.16
Introns	Cellular respiration	0.09	0.02, 0.17	0.04, 0.14
	Developmental growth	0.29	0.20, 0.38	0.23, 0.35
	Immune response	-0.08	-0.15, -3.40^{-3}	-0.13, -0.03
	Muscle tissue development	0.20	0.11, 0.29	0.14, 0.26
	Response to oxidative stress	0.37	0.28, 0.45	0.31, 0.42
Exons	Cellular respiration	0.19	0.10, 0.28	0.13, 0.25
	Developmental growth	-0.02	-0.10, 0.06	-0.07, 0.03
	Immune response	-0.14	-0.23, -0.05	-0.20, -0.08
	Muscle tissue development	0.01	-0.07, 0.08	-0.04, 0.06
	Response to oxidative stress	-0.07	-0.15, 0.02	-0.12, -0.02

Supplementary Table 8. Outputs of Bayesian GLMMs of annual mating success. Shown are point estimates, credible intervals (95% and 80%) and R^2 of the standardised β estimates of the six sexual traits, the total load, and age category. For the parameter 'age category', the β estimates of yearlings relative to adults are shown. The conditional R^2 refers to the variance explained by the fixed and random effects, whereas the marginal R^2 refers to the variance explained only by the fixed effects.

Approach	Predictor	Median	95% CI (lower, upper)	80% CI (lower, upper)	Conditional R2 [95%CI]	Marginal R2 [95%CI]
GERP	Lyre size	0.31	-0.07, 0.69	0.07, 0.57	0.55 [0.40, 0.69]	0.11 [0.02, 0.26]
	Eye comb size	0.13	-0.21, 0.48	-0.10, 0.35		
	Blue chroma	0.15	-0.05, 0.36	0.01, 0.29		
	Attendance	1.32	0.71, 2.01	0.91, 1.74		
	Fighting rate	-0.06	-0.33, 0.21	-0.24, 0.11		
	Centrality	-0.59	-0.93, -0.23	-0.81, -0.36		
	Total load	-0.12	-0.38, 0.11	-0.28, -0.03		
	Age category	-0.17	-1.09, 0.74	-0.78, 0.42		
SnpEff	Lyre size	0.33	-0.08, 0.72	0.07, 0.58	0.55 [0.41, 0.68]	0.10 [0.02, 0.25]
	Eye comb size	0.14	-0.21, 0.49	-0.09, 0.37		
	Blue chroma	0.15	-0.06, 0.36	0.02, 0.29		
	Attendance	1.32	0.72, 1.94	0.92, 1.73		
	Fighting rate	-0.06	-0.32, 0.20	-0.23, 0.11		
	Centrality	-0.57	-0.94, -0.22	-0.82, -0.34		
	Total load	-0.11	-0.38, 0.16	-0.27, 0.06		
	Age category	-0.15	-1.04, 0.79	-0.73, 0.45		

Supplementary Table 9. Outputs of Bayesian GLMMs of the six sexual traits. Shown are point estimates, credible intervals (95% and 80%) and R^2 values of the standardised β estimates of total GERP load and age category. One model was constructed for each of the sexual traits (see Methods for details). For the parameter 'age category', the β estimates of yearlings relative to adults are shown. The conditional R^2 refers to the variance explained by the fixed and random effects, whereas the marginal R^2 refers to the variance explained only by the fixed effects.

**Response**	**Predictor**	**Median**	**95% CI (lower, upper)**	**80% CI (lower, upper)**	**Conditional R2 [95%CI]**	**Marginal R2 [95%CI]**
Lyre size	Total GERP load	-0.03	-0.10, 0.04	-0.07, 0.02	0.88 [0.86, 0.89]	0.74 [0.72, 0.75]
	Age category	-1.77	-1.86, -1.68	-1.82, -1.72		
Eye comb size	Total GERP load	$-3.3e^{-3}$	-0.09, 0.09	-0.06, 0.05	0.57 [0.49, 0.63]	0.40 [0.34, 0.45]
	Age category	-1.31	-1.47, -1.16	-1.41, -1.21		
Blue chroma	Total GERP load	-0.03	-0.13, 0.08	-0.10, 0.04	0.42 [0.32, 0.50]	0.10 [0.06, 0.15]
	Age category	-0.67	-0.85, -0.49	-0.79, -0.55		
Attendance	Total GERP load	-0.10	-0.19, -0.01	-0.16, -0.05	0.42 [0.33, 0.50]	0.33 [0.27, 0.39]
	Age category	-1.15	-1.31, -0.99	-1.26, -1.05		
Fighting rate	Total GERP load	0.01	-0.09, 0.12	-0.06, 0.08	0.18 [0.09, 0.30]	0.07 [0.02, 0.12]
	Age category	-0.60	-0.83, -0.37	-0.75, -0.44		
Centrality	Total GERP load	0.04	-0.09, 0.16	-0.04, 0.12	0.44 [0.32, 0.55]	0.08 [0.03, 0.13]
	Age category	0.62	0.40, 0.84	0.48, 0.76		

Supplementary Table 10. Outputs of Bayesian GLMMs of the six sexual traits. Shown are point estimates, credible intervals (95% and 80%) and R^2 values of the standardised β estimates of total SnpEff load and age category. One model was constructed for each of the sexual traits (see Methods for details). For the parameter 'age category', the β estimates of yearlings relative to adults are shown. The conditional R^2 refers to the variance explained by the fixed and random effects, whereas the marginal R^2 refers to the variance explained only by the fixed effects.

Response	Predictor	Median	95% CI (lower, upper)	80% CI (lower, upper)	Conditional R2 [95%CI]	Marginal R2 [95%CI]
Lyre size	Total SnpEff load	0.04	-0.04, 0.10	-0.01, 0.08	0.88 [0.86, 0.89]	0.73 [0.72, 0.75]
	Age category	-1.77	-1.84, -1.69	-1.82, -1.72		
Eye comb size	Total SnpEff load	-0.01	-0.10, 0.07	-0.07, 0.04	0.57 [0.50, 0.63]	0.40 [0.35, 0.45]
	Age category	-1.31	-1.46, -1.15	-1.41, -1.21		
Blue chroma	Total SnpEff load	-0.07	-0.17, 0.04	-0.13, $9.80e^{-4}$	0.42 [0.33, 0.50]	0.11 [0.06, 0.15]
	Age category	-0.67	-0.84, -0.49	-0.79, -0.55		
Attendance	Total SnpEff load	-0.03	-0.11, 0.06	-0.08, 0.03	0.43 [0.34, 0.51]	0.32 [0.26, 0.38]
	Age category	-0.17	-1.32, -0.99	-1.27, -1.06		
Fighting rate	Total SnpEff load	-0.02	-0.12, 0.08	-0.09, 0.04	0.18 [0.09, 0.29]	0.07 [0.02, 0.12]
	Age category	-0.58	-0.82, -0.36	-0.74, -0.43		
Centrality	Total SnpEff load	-0.01	-0.14, 0.11	-0.09, 0.07	0.45 [0.32, 0.55]	0.08 [0.03, 0.12]
	Age category	0.62	0.40, 0.84	0.48, 0.76		

Supplementary Table 11. Outputs of Bayesian GLMMs of the direct and indirect effects of the total load on annual mating success (AMS). Shown are point estimates and credible intervals (95% and 80%) of the standardised β estimates of the direct and indirect effects of the total load on AMS through the six sexual traits calculated using the point method (see Methods for details). The model outputs for the regressions used to calculate the indirect effects are shown in Supplementary Tables 9–11. The direct effect of the total GERP load on AMS was estimated while correcting for all of the sexual traits.

Approach	Effect	Mediator	Median	95% CI (lower, upper)	80% CI (lower, upper)
GERP	Direct	–	-0.13	-0.36, 0.11	-0.29, 0.03
	Indirect	Lyre size	-0.01	-0.04, 0.01	-0.03, 0.01
		Eye comb size	0.00	-0.02, 0.02	-0.01, 0.01
		Blue chroma	0.00	-0.03, 0.01	-0.02, 0.01
		Attendance	-0.13	-0.28, -0.01	-0.22, -0.05
		Fighting rate	0.00	-0.02, 0.02	-0.01, 0.01
		Centrality	-0.02	-0.11, 0.05	-0.07, 0.02
SnpEff	Direct	–	-0.11	-0.38, 0.16	-0.27, 0.06
	Indirect	Lyre size	0.01	-0.01, 0.05	0.00, 0.03
		Eye comb size	0.00	-0.03, 0.02	-0.01, 0.01
		Blue chroma	-0.01	-0.04, 0.01	-0.03, 0.00
		Attendance	-0.03	-0.16, 0.09	-0.11, 0.04
		Fighting rate	0.00	-0.01, 0.02	-0.01, 0.01
		Centrality	0.01	-0.06, 0.09	-0.04, 0.06

Supplementary Table 12. Population differentiation among lekking sites. Shown above the diagonal are mean F_{ST} values, while the corresponding p-values are shown below the diagonal for each pairwise comparison.

Lekking site	Kummunsoa	Lehtusuo	Nyrölä	Saarisuo	Teerisuo
**Kummunsoa**	–	0.012	0.017	0.014	0.016
**Lehtusuo**	0.512	–	0.014	0.009	0.009
**Nyrölä**	0.527	0.457	–	0.017	0.017
**Saarisuo**	0.500	0.524	0.501	–	0.013
**Teerisuo**	0.488	0.506	0.536	0.527	–

Supplementary Table 13. Outputs of Bayesian linear models testing for differences in the total GERP and SnpEff loads and inbreeding (F_{ROH}) among leks. Shown are the median β estimates and their credible intervals (95% and 80%). Lekking site was used as a predictor, where Kummunsuo was the reference lek, and the response variable was z-transformed. Bold numbers indicate that the CIs do not overlap zero.

**Response**	**Lekking site**	**Median β estimate**	**95% CI**	**80% CI**
Total GERP load	Lehtusuo	-1.62	-0.61, 0.27	-0.43, 0.12
	Nyrölä	-0.10	-0.47, 0.26	-0.34, 0.13
	Saarisuo	-0.11	-0.53, 0.34	-0.40, 0.19
	Teerisuo	0.10	-0.38, 0.59	-0.23, 0.44
Total SnpEff load	Lehtusuo	-0.08	-0.17, 0.33	-0.09, 0.24
	Nyrölä	-0.03	-0.47, 0.41	-0.32, 0.25
	Saarisuo	-0.05	-0.41, 0.32	-0.30, 0.19
	Teerisuo	-0.17	-0.65, 0.33	-0.49, 0.16
F_{ROH}	Lehtusuo	-0.58	**-0.99, -0.16**	**-0.85, -0.30**
	Nyrölä	-0.10	-0.44, 0.26	-0.33, 0.12
	Saarisuo	-0.27	-0.71, 0.16	-0.55, 0.02
	Teerisuo	-0.09	-0.55, 0.39	-0.39, 0.21

Supplementary Table 14. Summary statistics of the B10k and the scaffolded black grouse reference genomes. N50 and N90 are the length in base pairs of the shortest contig whose length sum makes up 50% and 90% of the total genome size, respectively. L50 and L90 are the count of the smallest number of contigs whose length sum makes up 50% and 90% of the total genome size, respectively.

**Statistic**	**B10k genome**	**Scaffolded genome**
Total length (bp)	1,002,957,384	1,003,452,484
N50	5,658,217	69,550,540
L50	49	5
N90	500,392	12,704,504
L90	267	18
Number of scaffolds	26,930	21,979
Number of scaffolds > 1kbp	10,613	5,662
Largest scaffold (bp)	32,946,576	189,864,486
Number of gaps	10,955	15,906

Supplementary Table 15. Summary of the number of genes and mutations identified for each GO term. Shown are the number of genes associated with each GO term that contain deleterious mutations, separately for GERP and SnpEff. Also shown are the total number of deleterious mutations within those genes stratified across genomic regions (promoters, introns, exons) for each GO term separately for GERP and SnpEff. Because there were too few high impact SnpEff mutations in genes associated with androgen metabolic processes, we did not calculate the total load based on this subset of mutations.

GO term	GERP				SnpEff			
	**genes**	**promoters**	**introns**	**exons**	**genes**	**promoters**	**introns**	**exons**
Androgen metabolic process	8	9	56	14	1	1	0	1
Cellular respiration	111	84	542	231	18	7	8	15
Developmental growth	504	563	3,920	1,505	76	32	49	56
Immune response	635	12,674	62,605	3,460	102	1,554	1,504	2,764
Muscle tissue development	304	384	2,963	932	43	17	26	38
Response to oxidative stress	340	320	2,126	907	61	25	26	47

References

1. Höglund, J., Alatalo, R. V., Lundberg, A., RintamÎki, P. T. & Lindell, J. Microsatellite markers reveal the potential for kin selection on black grouse leks. *Proc. R. Soc. Lond. B Biol. Sci.* **266**, 813–816 (1999).
2. Chen, R. S. *et al.* Effects of hunting on genetic diversity, inbreeding and dispersal in Finnish black grouse (*Lyrurus tetrix*). *Evol. Appl.* **16**, 625–637 (2023).
3. Corrales, C. & Höglund, J. Maintenance of gene flow by female-biased dispersal of Black Grouse Tetrao tetrix in northern Sweden. *J. Ornithol.* **153**, 1127–1139 (2012).
4. Caizergues, A. & Ellison, L. N. Natal dispersal and its consequences in Black Grouse Tetrao tetrix: Natal dispersal and its consequences in Black Grouse. *Ibis* **144**, 478–487 (2002).
5. Warren, P. K. & Baines, D. Dispersal, survival and causes of mortality in black grouse Tetrao tetrix in northern England. *Wildl. Biol.* **8**, 91–97 (2002).
6. Manichaikul, A. *et al.* Robust relationship inference in genome-wide association studies. *Bioinformatics* **26**, 2867–2873 (2010).
7. Lebigre, C., Alatalo, R. V., Forss, H. E. & Siitari, H. Low levels of relatedness on black grouse leks despite male philopatry. *Mol. Ecol.* **17**, 4512–4521 (2008).
8. Lebigre, C., Alatalo, R. V. & Siitari, H. Female-biased dispersal alone can reduce the occurrence of inbreeding in black grouse (*Tetrao tetrix*). *Mol. Ecol.* **19**, 1929–1939 (2010).
9. Soulsbury, C. D., Alatalo, R. V., Lebigre, C. & Siitari, H. Restrictive mate choice criteria cause age-specific inbreeding in female black grouse, Tetrao tetrix. *Anim. Behav.* **83**, 1497–1503 (2012).
10. Davydov, E. V. *et al.* Identifying a High Fraction of the Human Genome to be under Selective Constraint Using GERP++. *PLoS Comput. Biol.* **6**, e1001025 (2010).

11. Cingolani, P. *et al.* A program for annotating and predicting the effects of single nucleotide polymorphisms, SnpEff. *Fly (Austin)* **6**, 80–92 (2012).
12. Booy, G., Hendriks, R. J. J., Smulders, M. J. M., Van Groenendael, J. M. & Vosman, B. Genetic Diversity and the Survival of Populations. *Plant Biol.* **2**, 379–395 (2000).
13. Hedrick, P. W. & Garcia-Dorado, A. Understanding Inbreeding Depression, Purging, and Genetic Rescue. *Trends Ecol. Evol.* **31**, 940–952 (2016).
14. Mathur, S., Tomeček, J. M., Tarango-Arámbula, L. A., Perez, R. M. & DeWoody, J. A. An evolutionary perspective on genetic load in small, isolated populations as informed by whole genome resequencing and forward-time simulations. *Evolution* **77**, 690–704 (2023).
15. The 1000 Genomes Project Consortium. A map of human genome variation from population-scale sequencing. *Nature* **467**, 1061–1073 (2010).
16. Sims, D., Sudbery, I., Ilott, N. E., Heger, A. & Ponting, C. P. Sequencing depth and coverage: key considerations in genomic analyses. *Nat. Rev. Genet.* **15**, 121–132 (2014).
17. Smeds, L. & Ellegren, H. From high masked to high realized genetic load in inbred Scandinavian wolves. *Mol. Ecol.* **32**, 1567–1580 (2023).
18. Huber, C. D., Kim, B. Y. & Lohmueller, K. E. Population genetic models of GERP scores suggest pervasive turnover of constrained sites across mammalian evolution. *PLOS Genet.* **16**, e1008827 (2020).
19. Eyre-Walker, A. & Keightley, P. D. The distribution of fitness effects of new mutations. *Nat. Rev. Genet.* **8**, 610–618 (2007).
20. Agarwal, I., Fuller, Z. L., Myers, S. R. & Przeworski, M. Relating pathogenic loss-of-function mutations in humans to their evolutionary fitness costs. *eLife* **12**, e83172 (2023).

21. Pagel, K. A. *et al.* When loss-of-function is loss of function: assessing mutational signatures and impact of loss-of-function genetic variants. *Bioinformatics* **33**, i389–i398 (2017).

22. Xu, Y.-C. & Guo, Y.-L. Less Is More, Natural Loss-of-Function Mutation Is a Strategy for Adaptation. *Plant Commun.* **1**, 100103 (2020).

23. Klim, J., Zielenkiewicz, U. & Kaczanowski, S. Loss-of-function mutations are main drivers of adaptations during short-term evolution. *Sci. Rep.* **14**, 7128 (2024).

24. MacArthur, D. G. *et al.* A Systematic Survey of Loss-of-Function Variants in Human Protein-Coding Genes. *Science* **335**, 823–828 (2012).

25. Zahavi, A. & Zahavi, A. *The Handicap Principle: A Missing Piece of Darwin's Puzzle*. (Oxford University Press, 1999).

26. Kurtz, J. & Sauer, K. P. The immunocompetence handicap hypothesis: testing the genetic predictions. *Proc. R. Soc. Lond. B Biol. Sci.* **266**, 2515–2522 (1999).

27. Costantini, D. The Oxidative Costs of a Colourful Life. in *The Role of Organismal Oxidative Stress in the Ecology and Life-History Evolution of Animals* 287–322 (Springer Nature Switzerland, Cham, 2024). doi:10.1007/978-3-031-65183-0_8.

28. Testosterone and male mating success on the black grouse leks. *Proc. R. Soc. Lond. B Biol. Sci.* **263**, 1697–1702 (1996).

29. Rintamaki, P. T. Combs and sexual selection in black grouse (*Tetrao tetrix*). *Behav. Ecol.* **11**, 465–471 (2000).

30. Kervinen, M., Alatalo, R. V., Lebigre, C., Siitari, H. & Soulsbury, C. D. Determinants of yearling male lekking effort and mating success in black grouse (*Tetrao tetrix*). *Behav. Ecol.* **23**, 1209–1217 (2012).

31. Ludwig, G. X., Alatalo, R. V., Helle, P. & Siitari, H. Individual and Environmental Determinants of Daily Black Grouse Nest Survival Rates at Variable Predator Densities. *Ann. Zool. Fenn.* **47**, 387–397 (2010).

32. Briffa, M. & Sneddon, L. U. Physiological constraints on contest behaviour. *Funct. Ecol.* **21**, 627–637 (2007).

33. Guderley, H. & Couture, P. Stickleback Fights: Why Do Winners Win? Influence of Metabolic and Morphometric Parameters. *Physiol. Biochem. Zool.* **78**, 173–181 (2005).

34. Zhu, M. & Zhao, S. Candidate Gene Identification Approach: Progress and Challenges. *Int. J. Biol. Sci.* 420–427 (2007) doi:10.7150/ijbs.3.420.

35. Barreiro, L. B. & Quintana-Murci, L. From evolutionary genetics to human immunology: how selection shapes host defence genes. *Nat. Rev. Genet.* **11**, 17–30 (2010).

36. Gilbert, R. & Uetz, G. W. Courtship and male ornaments as honest indicators of immune function. *Anim. Behav.* **117**, 97–103 (2016).

37. De La Peña, E. *et al.* Immune challenge of mating effort: steroid hormone profile, dark ventral patch and parasite burden in relation to intrasexual competition in male Iberian red deer. *Integr. Zool.* **15**, 262–275 (2020).

38. Moller, A. P., Christe, P. & Lux, E. Parasitism, Host Immune Function, and Sexual Selection. *Q. Rev. Biol.* **74**, 3–20 (1999).

39. Mandelker, L. Oxidative stress, free radicals, and cellular damage. *Stud. Vet. Med.* 1–17 (2011).

40. Zhang, G. Bird sequencing project takes off. *Nature* **522**, 34–34 (2015).

41. Feng, S., Stiller, J. & Deng. Dense sampling of bird diversity increases power of comparative genomics. *Nature* **587**, 252–257 (2020).

42. Luo, R. *et al.* SOAPdenovo2: an empirically improved memory-efficient short-read de novo assembler. *GigaScience* **1**, 18 (2012).

43. Xu, M. *et al.* TGS-GapCloser: A fast and accurate gap closer for large genomes with low coverage of error-prone long reads. *GigaScience* **9**, giaa094 (2020).

44. Putnam, N. H. *et al.* Chromosome-scale shotgun assembly using an in vitro method for long-range linkage. *Genome Res.* **26**, 342–350 (2016).

45. Camacho, C. *et al.* BLAST+: architecture and applications. *BMC Bioinformatics* **10**, 421 (2009).

46. Smit, A., Hubley, R. & Green, P. RepeatModeler Open. (2008).

47. Smit, A., Hubley, R. & Green, P. RepeatMasker Open. (2013).

48. Stanke, M., Diekhans, M., Baertsch, R. & Haussler, D. Using native and syntenically mapped cDNA alignments to improve *de novo* gene finding. *Bioinformatics* **24**, 637–644 (2008).

49. Korf, I. Gene finding in novel genomes. *BMC Bioinformatics* **5**, 59 (2004).

50. Dobin, A. *et al.* STAR: ultrafast universal RNA-seq aligner. *Bioinformatics* **29**, 15–21 (2013).

51. Cantarel, B. L. *et al.* MAKER: An easy-to-use annotation pipeline designed for emerging model organism genomes. *Genome Res.* **18**, 188–196 (2008).

52. Chan, P. P., Lin, B. Y., Mak, A. J. & Lowe, T. M. tRNAscan-SE 2.0: improved detection and functional classification of transfer RNA genes. *Nucleic Acids Res.* **49**, 9077–9096 (2021).

53. Purcell, S. *et al.* PLINK: A Tool Set for Whole-Genome Association and Population-Based Linkage Analyses. *Am. J. Hum. Genet.* **81**, 559–575 (2007).

54. Wickham, H. *et al.* Welcome to the Tidyverse. *J. Open Source Softw.* **4**, 1686 (2019).

55. Danecek, P. *et al.* The variant call format and VCFtools. *Bioinformatics* **27**, 2156–2158 (2011).

56. Canty, A. & Ripley, B. D. boot: Bootstrap R (S-Plus) functions. (2021).

57. Humble, E., Paijmans, A. J., Forcada, J. & Hoffman, J. I. An 85K SNP Array Uncovers Inbreeding and Cryptic Relatedness in an Antarctic Fur Seal Breeding Colony. *G3 GenesGenomesGenetics* **10**, 2787–2799 (2020).

58. Korneliussen, T. S. & Moltke, I. NgsRelate: a software tool for estimating pairwise relatedness from next-generation sequencing data. *Bioinformatics* btv509 (2015) doi:10.1093/bioinformatics/btv509.

59. Waples, R. K., Albrechtsen, A. & Moltke, I. Allele frequency-free inference of close familial relationships from genotypes or low-depth sequencing data. *Mol. Ecol.* **28**, 35–48 (2019).

60. Brooks, Mollie, E. *et al.* glmmTMB Balances Speed and Flexibility Among Packages for Zero-inflated Generalized Linear Mixed Modeling. *R J.* **9**, 378 (2017).

61. Young, D. S. **tolerance** : An *R* Package for Estimating Tolerance Intervals. *J. Stat. Softw.* **36**, (2010).

62. Carbon, S. & Mungall, C. Gene Ontology Data Archive. Zenodo https://doi.org/10.5281/ZENODO.10536401 (2024).

63. Carbon, S. *et al.* AmiGO: online access to ontology and annotation data. *Bioinformatics* **25**, 288–289 (2009).

64. Aourir, M., Znari, M., El Abbassi, A. & Radi, M. Growth Patterns and Developmental Strategy in the Black-Bellied Sandgrouse *Pterocles orientalis*. *Ardeola* **63**, 311–327 (2016).

65. Soulsbury, C. D., Siitari, H. & Lebigre, C. Stabilising selection on immune response in male black grouse Lyrurus tetrix. *Oecologia* **186**, 405–414 (2018).

66. Hämäläinen, A., Alatalo, R. V., Lebigre, C., Siitari, H. & Soulsbury, C. D. Fighting behaviour as a correlate of male mating success in black grouse *Tetrao tetrix* . *Behav. Ecol. Sociobiol.* **66**, 1577–1586 (2012).

67. Rintamäki, P. T., Höglund, J., Alatalo, R. V. & Lundberg, A. Correlates of male mating success on black grouse (Tetrao tetrix L.) leks. *Ann. Zool. Fenn.* **38**, 99–109 (2001).

68. Royle, N. J., Orledge, J. M. & Blount, J. D. Early Life-History Effects, Oxidative Stress, and the Evolution and Expression of Animal Signals. in *Animal Signaling and Function* (eds. J. Irschick, D., Briffa, M. & Podos, J.) 11–46 (Wiley, 2015). doi:10.1002/9781118966624.ch2.
